# Urban Green Space Perception and Its Contribution to Well-Being

**DOI:** 10.3390/ijerph14070766

**Published:** 2017-07-12

**Authors:** Gyula Kothencz, Ronald Kolcsár, Pablo Cabrera-Barona, Péter Szilassi

**Affiliations:** 1 Department of Geoinformatics—Z_GIS, University of Salzburg, Schillerstrasse 30, 5020 Salzburg, Austria; pablo.cabrera-barona@stud.sbg.ac.at; 2 Department of Physical Geography and Geoinformatics, University of Szeged, Egyetem utca 2-6, 6722 Szeged, Hungary; kolcsarrony@hotmail.com (R.K.); toto@geo.u-szeged.hu (P.S.)

**Keywords:** urban green spaces, perceived green space characteristics, perceived well-being benefits, quality of life, crowd-sourced geo-tagged data, questionnaire survey

## Abstract

Individual perceptions are essential when evaluating the well-being benefits from urban green spaces. This study predicted the influence of perceived green space characteristics in the city of Szeged, Hungary, on two well-being variables: the green space visitors’ level of satisfaction and the self-reported quality of life. The applied logistic regression analysis used nine predictors: seven perceived green space characteristics from a questionnaire survey among visitors of five urban green spaces of Szeged; and the frequency of green space visitors’ crowd-sourced recreational running paths and photographs picturing green space aesthetics. Results revealed that perceived green space characteristics with direct well-being benefits were strong predictors of both dependent variables. Perceived green space characteristics with indirect, yet fundamental, well-being benefits, namely, regulating ecosystem services had minor influence on the dependent variables. The crowd-sourced geo-tagged data predicted only the perceived quality of life contributions; but revealed spatial patterns of recreational green space use and aesthetics. This study recommends that regulating ecosystem services should be planned with a focus on residents’ aesthetic and recreational needs. Further research on the combination of green space visitors´ perceptions and crowd-sourced geo-tagged data is suggested to promote planning for well-being and health benefits of urban green spaces.

## 1. Introduction

Societal benefits supplied by urban green spaces (UGS) to city dwellers are vital to maintain and increase urban citizens’ quality of life (QoL) [1,2]. UGS are essential in mitigating high summer temperatures of their grounds and nearby surroundings [3,4], and are vital in air pollution removal and noise abatement [5,6]. They are also highly regarded for positive effects in promoting their visitors’ physical and mental health [7,8,9] and providing opportunities for social interactions and recreation [9,10,11]. The QoL benefits derived from UGS are increasingly central to urban society [12,13] and, therefore, understanding visitors’ attitudes and perceptions of UGS is essential for informed urban planning [14,15]. However, exploring visitors’ perceptions of green spaces is challenging as it depends on cognitive, affective, and behavioural components and, therefore, sensory perceptions are individually different [16,17,18,19].

Consequently, sensory dimensions and experiential contacts with the environment are crucial building blocks of area-based perceptions [20,21], and subjective well-being is driven by location-specific environmental endowments [22]. For example, increasing neighbourhood green space ratio proved to have a positive effect on residents’ self-reported life satisfaction in Australian capital cities [23]. Hence, the relation between UGS visitors’ perception and green space characteristics is a key factor to understanding the mechanism of how tangible and intangible benefits are derived from ecosystems supplied by UGS [24]. Existing research varies from a focus on a particular UGS function to a set of services. For example, Nasution and Zahrah [25] linked community perceptions of public open spaces to QoL in Medan, Indonesia, and Oliveira, et al. [26] linked visitors’ preferences of UGS to their perceived cooling effect of UGS in Lisbon, Portugal. The association of perceived well-being benefits to a range of ecosystem services provided by UGS and urban green infrastructure has been investigated by several scholars [27,28,29]. In addition, a mediation effect of perceived restorativeness was found between settings of experiential green space contact (perception of nature, length of visit, and level of biodiversity) and self-reported well-being [30,31]. These studies provide the context for the first pillar of our research: visitors’ level of satisfaction and perceived QoL contribution of UGS are key individual-level measures that are subjectively affected by area-based green space characteristics.

The second pillar of our study centres on the possible suitability of frequency of crowd-sourced geo-tagged data, recorded within UGS, to gauge visitors’ perception of UGS. Scholars who have researched the sensory dimensions of UGS have tended to use conventional data sources to identify associations and causalities in the human attitude towards UGS [32]. Therefore, sources of information are based on traditional data acquisition methods such as questionnaire surveys [15,33,34]. For example, Baur et al. [16] used randomly-distributed mail questionnaires to capture green space users’ and non-users’ attitudes toward urban nature parks in Portland, OR, USA. Questionnaire surveys were also used by Lo and Jim [34] to assess preferences of Hong Kong citizens for UGS.

Despite the successful application of questionnaire-based analytical methods, it is timely to test the capabilities of data crowd-sourcing to understand visitors’ attitudes towards UGS. The authors’ decision to use crowd-sourced data was three-fold. Firstly, UGS visitors of recent years can use location-aware technologies to spatially log their activities; for example, UGS visitors can geo-tag their photographs that they take during their UGS visits, or log trajectories of their physical activities. Second, geo-tagged user-generated content (UGC), a form of Big Data, is able to expose patterns and relationships which would be hidden from questionnaire-based methods [32,35]. Third, geo-located and voluntarily gifted UGC has already demonstrated potential in other fields of geographic science [36,37,38]. For example, Feick and Robertson [39] used geo-tagged photographs to explore spatial expressions of urban places, and geo-tagged photographs helped Richards and Friess [40] to understand cultural ecosystem services supplied by mangroves in Singapore. Santos, et al. [41] used GPS track-logs recorded by mountain bikers and runners in a UGS of Lisbon, Portugal, to present spatial overlap and potential conflicts of interest in recreational trail use. Due to their intangible nature, cultural ecosystem services, such as aesthetic appreciation, and recreational capacity are challenging to quantify [17,42]. Therefore, scholars increasingly utilise geo-tagged UGC to evaluate aesthetic enjoyment [40,43] and recreation [41,44] as their volume and spatial patterns can indicate recreational capacity and aesthetic appreciation [40,45,46,47]. Furthermore, the InVEST ecosystem modelling tool implements geo-tagged UGC to evaluate cultural ecosystem services [48]. Dunkel [49] also suggests that geo-tagged UGC “may contribute to a more balanced assessment of the perceived landscape”. Therefore, our assumption is that the frequency of geo-tagged user entries should enable us to gauge visitors’ perceptions of UGS.

The present study aimed to support urban planners and green space managers from the city of Szeged, Hungary, with information on the role of the city UGS in citizens’ QoL. Therefore, expert interviews were conducted with urban planners and environment managers of the Szeged City Council to assess their needs. Their interest was to understand how green space characteristics influence the use and perception of the city green spaces. The study was designed and conducted accordingly and aimed to answer the following two research questions:

- Research Question 1: To what extent did perceived green space characteristics influence visitors’ levels of satisfaction with the visited green spaces and the self-reported QoL contributions of the study areas?
- Research Question 2: Was the frequency of geo-tagged photographs and running trajectories statistically related to visitors’ perception of UGS?

The aims of the study were:

- To reveal the role of green space characteristics in visitors’ self-reported levels of satisfaction and the perceived QoL contribution of the studied green spaces.
- To statistically explore the potential of frequency of geo-tagged photographs and running trajectories in revealing human perceptions of UGS.

## 2. Materials and Methods

### 2.1. Study Area

**Figure 1:**
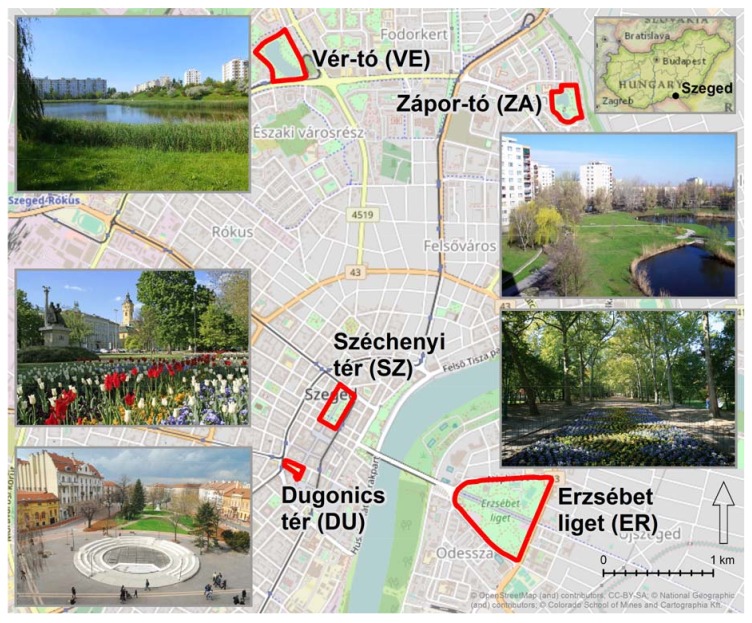
Location of the study areas. Figure created by the authors. Sources of base maps: [50,51,52].

The study areas were five urban green spaces (UGS) within the city of Szeged (Figure 1), selected to represent diversity and comparability; Erzsébet liget (ER), Dugonics tér (DU), Széchenyi tér (SZ), Vér-tó (VE), and Zápor-tó (ZA). ER, with a very high proportion of lawn and tree patches, is in close proximity to the city centre and attracts mostly recreational visitors and is occasionally allocated to public events. DU, completely refurbished in 2014, is located in the scenic city centre and boasts massive pedestrian traffic. Approximately 50% of the area is vegetated, which provides suitable shelter in the city centre. The picturesque SZ is the central square of the city and is surrounded by aesthetic architecture and is very popular with both locals and tourists. VE is located in a massive residential area characterised by non-attractive 5–10 storey blocks of houses and is affected by serious noise pollution from adjacent roads. Similarly, ZA is located in a residential area and is favoured by nearby residents for its peacefulness and its high percentage of green cover.

### 2.2. Data Sources

#### 2.2.1. Questionnaire Survey

A survey was conducted with visitors of the five UGS to examine attitudes towards the study areas. Thirty questions evaluated the perceived quality of life (QoL) contributions, and the answers were measured on a Likert scale (1–5; 1—very dissatisfied; 5—very satisfied). The details of the survey were reported by Kothencz and Blaschke [53]. At the survey the exact location (settlement and street) of respondents’ residency had been asked, and exclusively Szeged residents’ records were considered for further investigation. After data validation, 227 completed questionnaires were used. Answers for nine questions were used as dependent or independent variables in the analysis (Section 2.3) (Table 1). Each of these questions was informed by relevant literature (see Table 1). This ensured that the seven perceived green space characteristics, selected as independent variables, are in line with experiences of urban ecosystem attitude studies and are able to capture perceptions of ecosystem services that are essential for the quality of urban life.

#### 2.2.2. Running Trajectories

**Figure 2:**
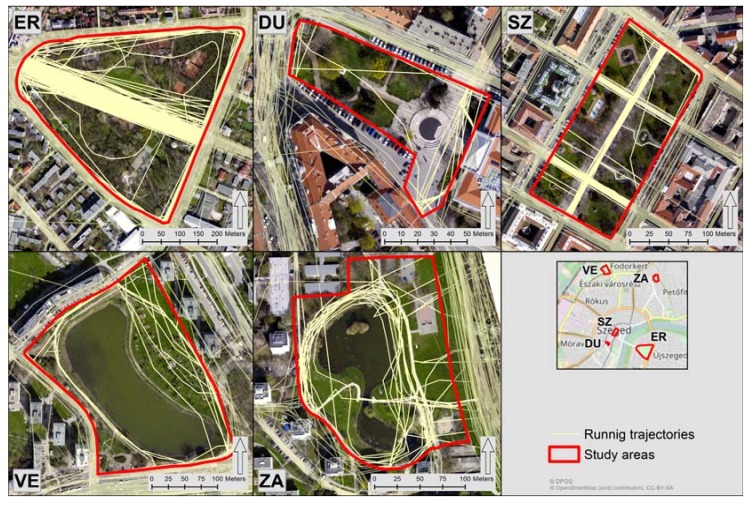
Running trajectories touching or crossing the grounds of the study areas. ER: Erzsébet liget; DU: Dugonics tér; SZ: Széchenyi tér; VE: Vér-tó; ZA: Zápor-tó. Figure created by the authors. Sources of base maps: [51,59].

Running trajectories are the ground paths of runners as they move through space. All running trajectories available on the crowd-sourced running path sharing website, Futótérkép [58], for Szeged, were extracted in April 2016. Trajectories touching or crossing the grounds of the five study areas were selected and imported to a polyline GIS layer (Figure 2).

Trajectories running through those parts of the UGS where they were not supposed to occur (e.g., through a lake; see VE for example) were not deleted, as they also counted as a logged path even though the GPS receiver of the runner was not very accurate. The number of running trajectories (NoOfTrks) per UGS were added together and used in the subsequent analysis (Section 2.3) as independent variables representing an objective recreational use of the study areas (Table 2). The design of this variable was supported by relevant literature in which trajectory-based geo-tagged UGC proved to indicate objective recreational use of public open spaces [41,44,60].

#### 2.2.3. Green Space Photographs

**Figure 3:**
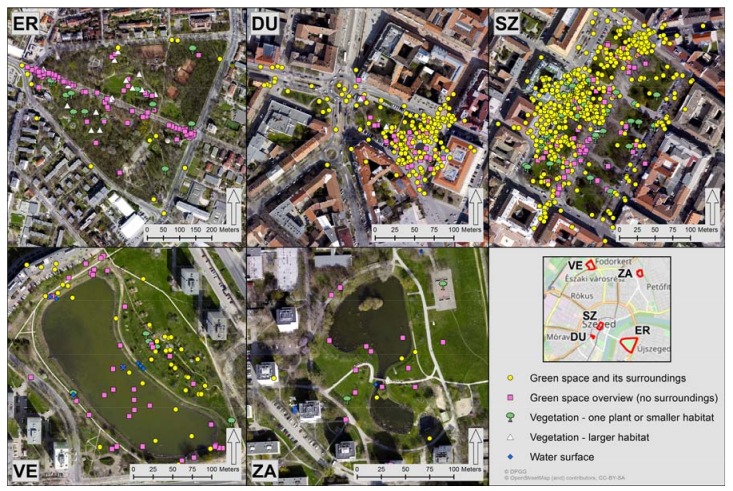
Distribution of images picturing green space aesthetics. ER: Erzsébet liget; DU: Dugonics tér; SZ: Széchenyi tér; VE: Vér-tó; ZA: Zápor-tó. Figure created by the authors. Sources of base maps: [51,59].

Photographs geo-located on the grounds of the UGS or within their one-block-wide perimeter were downloaded from the photo sharing website Panoramio [61] and input to a GIS as point features. The one-block-wide perimeter was needed to assure the inclusion of those green space photographs which were either incorrectly geo-located by users' cameras or were uploaded to incorrect locations by the photographer. Seventeen categories of image themes were formed based on a preliminary visual examination of each photograph. Then a thorough visual content analysis was conducted and each photograph was assigned to one of the seventeen classes. Guidance for the category development and content analysis was drawn from previous work [62,63,64]. Photographs were deliberately not distinguished based on whether they were taken by locals or tourists, as residents and tourists do not hold significantly different place perceptions [65]. Photographs relevant to more than one category were assigned to the class of which the dominant content of the image related the most. Photographs in twelve classes pictured non-green space aesthetic-related content, such as people, events, animals, and archive photographs of the UGS. Five of the seventeen categories were designed to accommodate images with green space aesthetics (Figure 3).

The number of photographs in the five categories imaging green space aesthetics were summed up for each UGS. The proportion of images picturing green space aesthetics (AesthPct) to the total number of photographs per UGS were calculated for each study area. The percentages per UGS were used as independent variables representing aesthetic appreciation of the study areas (Table 3).

### 2.3. Regression Analysis

Each row of the table inputted in the analysis represented individual-level data; the questionnaire record of a single respondent. The unique identification code of each park, of which the particular person responded, was added to the records. The total number of running trajectories per the relevant UGS and the proportion of images picturing green space aesthetics per the relevant UGS were also added to every row of the input table.

Ordinal logistic regressions were applied using two dependent variables representing individual-level perceptions of the UGS: visitors’ level of satisfaction with the UGS and the perceived QoL contribution of the UGS. For each respondent, individual regressions were performed.

Ordinal logistic regressions are logistic generalized linear models (GLMs) where the dependent variable is a categorical ordered variable. GLMs can be expressed as follows (Equation (1)):

(1) $$P\left(Y\right)=logit\left(b_{0}+b_{1}x_{1}+b_{2}x_{2}+\ldots +b_{n}x_{n}\right)$$

where *Y* represents the categorical ordered variable, and *x* and *b* represent the independent variables and their coefficients, respectively.

For each dependent variable two models were performed. One model included the seven perceived green space characteristics as subjective area-based independent variables from the questionnaire: the perception of nature, noise abatement, capacity for recreation, microclimate regulation, habitat, air purification, and the visual appearance.

In the second model two variables extracted from the crowd-sourced geo-tagged data were added to the subjective independent variables. These were the proportion of images picturing green space aesthetics and the number of running trajectories, representing objective green space popularity through quantitative data. Therefore, their values for each UGS were assigned to the records of the seven subjective area-based independent variables.

## 3. Results

The results of the logistic analyses are shown in Table 4 with statistically significant variables highlighted. Results for Model 1 are explained in detail to support the interpretation of all the other models.

The odds ratios obtained from Model 1 can be interpreted as follows:

1. The relative odds of experiencing a very positive satisfaction with the green spaces are 1.66 times greater for respondents who perceived high degree of nature in the study areas than for those who did not.
2. For survey participants perceiving high recreational capacity of the green spaces, the relative odds of experiencing very positive green space satisfaction are 1.47 times greater than for those that reported poor recreational capacity.
3. The relative odds of experiencing positive satisfaction with the green spaces are 1.89 times greater for respondents with a high aesthetic satisfaction of the green spaces than for those who perceived low aesthetics.

Interpretation of the Akaike information criterion (AIC): Considering the four models, Model 1 had the best performance indicated by the lowest AIC value. If models only with questionnaire-based variables are concerned, still Model 1 performed better. If the extended models are compared Model 2 performed better.

## 4. Discussion

The present study fulfilled two goals. Firstly, it uncovered the role of perceived green space characteristics in visitors’ level of satisfaction and the perceived quality of life (QoL) contribution. Secondly, the research demonstrated the potential of frequency of geo-tagged photographs and running trajectories in revealing human perceptions of urban green spaces (UGS).

### 4.1. Answer to Research Question 1

The results demonstrated the extent to which the seven perceived green space characteristics influenced visitors’ levels of satisfaction and the self-reported QoL. Every model revealed that visual appearance of the UGS was the most crucial predictor of visitors’ levels of satisfaction and the self-reported QoL (at 1% level of significance in Models 1, 2 and 4; at 5% level of significance in Model 3). The perception of nature was the second most influential green space characteristic that highly affected visitors’ satisfaction in Models 1 and 2 already at 1% level of significance. The third most crucial green space characteristic was the perceived recreational capacity. This positively influenced visitors’ level of satisfaction with the UGS in Models 1 and 2 at 5% level of significance, and the perceived QoL contribution of the five UGS in Model 4 at 5% level of significance. Of lesser importance, habitat contributed to visitors’ levels of satisfaction in Models 1 and 2 at 10% level of significance. Another notable perceived green space characteristic, the microclimate regulation, affected visitors’ self-reported QoL in Model 3 at 10% level of significance.

Our work identified that regulating ecosystem services were largely underrated by survey participants. Noise abatement, microclimate regulation and air purification services supplied by the study areas seemed to have only minor significance on visitors’ perceptions. Likewise, habitat was underappreciated. There are two possible arguments to explain these results. The first is the mainly ornamental purpose of Szeged’s city centre green spaces means they are likely to contribute less to regulating ecosystem services than those which are located away from the city centre. The second possible reason stems from a lack of societal awareness of the fundamental importance of regulating ecosystem services, in that the benefits derived from regulating services are not directly “tangible” or perceivable. Even though cultural ecosystem services such as the scenery and the recreation are also intangible, green space visitors can directly benefit from them through aesthetic appreciation, relaxation and physical activity. Therefore, visitors feel more attached to cultural services than regulating or habitat services. These findings are confirmed by results of earlier studies. Tyrväinen, et al. [28] found recreational opportunities supplied by UGS and their contribution to pleasant cityscape more influential to survey participants than noise abatement, dust removal, or ameliorating climatic conditions. Survey participants in Bilbao, Spain, rated regulating ecosystem services of lower importance than cultural services from which they directly derive well-being benefits [66]. They also found a lack of societal awareness of ecosystems’ capacity to benefit society. The very positive contribution of perception of nature to visitors’ level of satisfaction is a welcome addition to the findings of our study. This may be explained by perceived restorativeness mediating the effect of the experienced nature qualities on visitors’ level of satisfaction [31,67].

### 4.2. Answer to Research Qusetion 2

Our findings suggest that visitors' perception of the UGS were effectively conveyed through the seven questionnaire items, while frequencies of geo-tagged photographs and running trajectories were only partly related to individual-level perceptions of the study areas. The number of running trajectories and the proportion of images picturing green space aesthetics were found significant at 1% and 5% level of significance, respectively, in relation to the perceived QoL contribution of the UGS in Model 4. However, the two crowd-sourced area-based variables were not strong enough to support survey participants’ satisfaction with the study areas. Consequently, the potentials of frequency of geo-tagged photographs and running trajectories were limited in revealing human perceptions towards the studied UGS. On the other hand, geo-tagged photographs and running trajectories exposed spatial patterns in their distribution. As it can be seen in Figure 3, the spatial concentration of geo-tagging locations of photographs in ER, DU, and SZ are immediately identifiable. The same is true for the concentration of running trajectories in ER (Figure 2).

The findings of our work are in line with those from previous studies by showing that Big Data, in the present case geo-tagged UGC, is suitable to identify general relations [35,43], while traditional data sources, such as variables from a questionnaire survey, are able to reveal causalities [32]. The findings also support other studies on trajectory and photography-based crowd-sourced geo-tagged data that indicate recreational capacity and aesthetic appreciation of public open spaces [40,43,44,60].

### 4.3. Planning and Management Implications

#### 4.3.1. Planning Regulating Ecosystem Services in Line with Residents’ Aesthetic and Recreational Needs

Aesthetics are a contextual feature affecting personal perspectives and have implications for landscape and ecological planning and intervention [68]. Therefore, from a planning perspective an UGS providing regulating ecosystem services such as microclimate regulation or air purification might be more appreciated by visitors if it also offers high aesthetic enjoyment. Physical well-being is often achieved through recreational activities in green spaces [21], and according to our results recreational opportunities offered by UGS are crucial to their visitors.

Hence, it is vital that urban planning and management implement development of regulating ecosystem services with a consideration of cultural services in their strategic and operational goals. In addition, education is needed to raise environmental awareness of intangible ecosystem services as this would enable a better appreciation of them as crucial sources of our well-being benefits [66].

#### 4.3.2. Combined Use of UGS Visitors’ Perceptions and Their Crowd-Sourced Geo-Tagged Data

There are interventions in green space planning and management that require qualitative knowledge, for example, to understand visitors’ perception of the scenery of the UGS. To achieve this goal, questionnaire-based methods are still required. However, green space managers need to be aware of the measured green space usage and appreciation, and this is the specific area where geo-tagged UGC can contribute valuable information to help management decisions. Frequency and spatial concentration of running trajectories may function as measured recreational green space usage [41], whilst the spatial concentration of geo-tagging locations of visitors’ photographs can predict environmental aesthetics that attract green space visitors [69].

Even though geo-tagged UGC has potential planning implications [49,69,70], currently there is no evidence that urban green space managers extensively use questionnaire data in conjunction with UGS visitors’ geo-tagged data. The authors recommend that scientific communities further investigate a mixed-methods approach; a collective application of questionnaire-based variables and crowd-sourced geo-tagged data on the use and aesthetic appreciation of UGS. When technically matured, such an approach could lead to better evidence-based decisions in urban planning and management.

### 4.4. Future Outlook

Prior to the work the authors assessed the needs of urban planners and environment managers to understand residents’ perception of well-being benefits they derive from UGS. Future studies will follow in which urban planners and green space managers will be involved in all phases of the work, from the study design to the data acquisition and analysis to the result implementation. This would guarantee the full compatibility of the methodology and the results in decision support practices.

Individual-level objective variables, such as respondents’ socio-economic status, affect individual perceptions often leading to very different self-reported satisfaction [71,72]. Therefore, individual circumstances can be also incorporated in subsequent analyses. This would allow the authors and city councils to understand how perceived green space characteristics affect the satisfaction and well-being of various societal groups. In addition to the ordinal logistic regressions applied here, future studies could perform multi-level models in order to identify contextual effects of area-level characteristics of the green spaces on individual-level QoL and health.

The authors conducted the study as a pilot in Szeged. As the methodology is easily transferable to other cities, the authors would like to pilot it in other urban areas to gain more experience and possibly detect differences in preferences in perceived well-being benefits derived from urban green spaces.

The present study can be considered as a basis to understand the city as a complex system. The methods applied here for UGS can be transferred to the city scale. Future research may apply methodologies that go beyond green spaces and use questionnaires and crowd-sourced geo-tagged data for a better comprehension of other urban resources and services.

## 5. Conclusions

This study used answers from a questionnaire survey together with the frequency and spatial distribution of crowd-sourced geo-tagged images and running trajectories of five UGS in the city of Szeged, Hungary. The aim was to predict the influence of perceived green space characteristics, reported by surveyed green space visitors, on two well-being variables: visitors’ levels of satisfaction with the green spaces and the perceived quality of life contribution of the respective area. The ultimate goal was to inform urban planners and green space managers of Szeged about the effects of green space characteristics on well-being benefits that the citizens obtain from UGS. Four models of ordinal logistic regression analysis were applied. They disclosed that perceived green space characteristics with direct well-being benefits strongly influenced visitors’ green space attitude. The two well-being variables were particularly dependent on the perceived scenery of the study areas. Recreational capacity, which has direct well-being benefits, was also able to predict the two dependent variables. Perception of nature, an indirect well-being benefactor, was a strong predictor of visitors’ levels of satisfaction with the green spaces. Perception of green space characteristics referring to vital regulating ecosystem services with provision of indirect well-being benefits had a low influence on green space visitors’ levels of satisfaction and self-reported quality of life contribution of the study areas. The two crowd-sourced area-based variables, the geo-tagged photographs and running trajectories, were able to explain the perceived quality of life contribution of the green spaces but failed to explain visitors’ green space satisfaction. In turn, the geo-tagged UGC revealed fine-scaled spatial patterns within the study areas which highlighted the site popularity and real green space usage.

The following conclusions can be drawn for urban planning and management: Consideration of site aesthetics and recreational services is crucial for UGS development, and regulating ecosystem services should be planned accordingly. Awareness of the importance of regulating ecosystem services should be raised by environmental education. The joint use of questionnaire surveys, delivering perceptual information on UGS, and geo-tagged UGC, revealing real recreational green space usage and aesthetic appreciation, is recommended for further investigation. When technically ready, the approach could promote informed decisions in urban planning and management. This study has been carried out in close collaboration with the administration of the city of Szeged and the results will be presented to the relevant administrative units in order to assure citizens’ needs-centred planning.

## Figures and Tables

**Table 1:** Dependent and independent variables.

Questions Used from the Survey	Dependent or Independent Variable	Relevant Literature
How much do you like the area?	Level of satisfaction with the green space (LikeArea) [DV]	
How do you rate the quality of life here?	Perceived quality of life contribution of the green space (QoLRate) [DV]	
How natural do you think the area is?	Perception of nature (Nature) [IV]; [S]	[14,27]
How quiet is the area in terms of traffic noise?	Perceived noise abatement (Quietness) [IV]; [R]	[54,55]
How much does the area satisfy the function: Recreation?	Perceived capacity for recreation (Recreation) [IV]; [C]	[24,25]
How much does the area satisfy the function: Cooler summer temperatures provided by the green space?	Perceived microclimate regulation (HeatMitiga) [IV]; [R]	[26]
How much does the area satisfy the function: Shelter for a variety of plant and animal life?	Perceived habitat (Habitat) [IV]; [S]	[14,27,56]
How much does the area satisfy the function: Reduction of air pollution?	Perceived air purification (AirPollMit) [IV]; [R]	[28,29]
How much does the area satisfy the function: Visual appearance?	Visual appearance (Scenery) [IV]; [C]	[21,57]

DV: Dependent Variable; IV: Independent Variable; Reference to perception of regulating [R], cultural [C] or supporting [S] ecosystem service.

**Table 2:** Number of running trajectories.

Category	ER	DU	SZ	VE	ZA
Number of running trajectories	498	29	142	27	32

ER: Erzsébet liget; DU: Dugonics tér; SZ: Széchenyi tér; VE: Vér-tó; ZA: Zápor-tó.

**Table 3:** Number and proportion of images per UGS picturing green space aesthetics.

Category	ER	DU	SZ	VE	ZA
Green space and its surroundings	15	199	547	42	5
Green space overview (no surroundings)	77	34	68	42	15
Vegetation—one plant or smaller habitat	13	0	47	6	2
Vegetation—larger habitat	16	2	1	0	0
Water surface	0	0	0	7	1
Sum of images picturing green space aesthetics	121	235	663	97	23
Total number of images	210	360	965	179	30
Proportion of images picturing green space aesthetics (%)	57.6	65.3	68.7	54.2	76.7

**Table 4:** Results of the ordinal logistic regressions.

Dependent Variable	Model	Variable	Odds Ratio	Lower 95% CI	Upper 95% CI	AIC
LikeArea	Model 1	Nature ^+^	1.66	1.19	2.35	364.27
		Quietness	0.95	0.70	1.30	
		Recreat	1.47	1.01	2.18	
		HeatMitiga	0.92	0.65	1.31	
		Habitat *	1.38	0.98	1.97	
		AirPollMit	1.07	0.74	1.53	
		Scenery ^+^	1.89	1.30	2.78	
	Model 2	Nature ^+^	1.66	1.18	2.35	366.73
		Quietness	0.92	0.66	1.30	
		Recreat	1.48	1.01	2.20	
		HeatMitiga	0.89	0.62	1.26	
		Habitat *	1.40	0.99	2.00	
		AirPollMit	1.02	0.70	1.48	
		Scenery ^+^	1.97	1.34	2.95	
		AesthPct	1.00	0.96	1.04	
		NoOfTrks	1.00	0.99	1.00	
QoLRate	Model 3	Nature	1.04	0.75	1.43	430.12
		Quietness	1.14	0.84	1.54	
		Recreat	1.35	0.93	1.97	
		HeatMitiga *	1.33	0.96	1.87	
		Habitat	0.85	0.60	1.20	
		AirPollMit	1.33	0.94	1.88	
		Scenery	1.56	1.09	2.22	
	Model 4	Nature	1.04	0.75	1.46	411.56
		Quietness	0.93	0.67	1.36	
		Recreat	1.49	1.01	2.20	
		HeatMitiga	1.14	0.81	1.71	
		Habitat	0.89	0.62	1.21	
		AirPollMit	1.23	0.85	1.80	
		Scenery ^+^	1.74	1.20	2.58	
		AesthPct	1.05	1.01	1.02	
		NoOfTrks ^+^	1.00	1.01	1.02	

Variables in boldface are significant at the 5% level. There are variables in boldface that are also significant at the 1% level (^+^). There are additional variables that are significant at the 10% level (*). CI: Confidence interval; AIC: Akaike information criterion.
